# Reports of Cases Occurring in the Medical Ward of the Buffalo Hospital

**Published:** 1857-09

**Authors:** A. W. Nichols

**Affiliations:** Assistant to Chair of Clinical Medicine, University of Buffalo


ART. IV.—*Reports of Cases treated in the Male Medical Ward of the Buffalo Hospital of the Sisters of Charity*, AUSTIN FLINT, M. D., *Attending Physician, during the College Session of* 1856–7, *being from October* 1, 1856 *to February* 18, 1857. Reported by A. W. NICHOLS, M. D., Assistant to Chair of Clinical Medicine, University of Buffalo.

No. VI. *Apoplexy and Paralysis; Pseudo-apoplexy, arcus senilis.*

We lay before the reader, an analysis of several cases of apoplexy followed by hemiplegia, inviting attention to several interesting points in the apoplectic attack and in the paralytic condition which followed it. We introduce, also, a case of pseudo-apoplexy with arcus senilis, in which there was suspected fatty degeneration of the heart.

*Condition pior to attack; and mode of attack.*

No. I, aged 40, not ascertained; paralyzed when admitted; right side affected.

No. II, aged 43, laborer; had received an injury on the head four years ago; skull probably fractured; one year since had an affection of the brain, was delirious thirteen days; since then, has been subject to pain in the head and has not regained his strength; last winter, suffered from an attack of rheumatism; during present summer, has been exposed to the sun, much of the time; was attacked whilst walking in the sun, with paralysis of left upper extremity; in a short time he became unconscious; remained so a day or more; on recovering, found himself hemiplegic. Left side affected; corrugator and orbicular muscles of left eye paralyzed; intercostal muscles of left side motionless. Sensibility not materially affected; paralyzed limbs seem to him to be increased in size.

No. III, aged 54, laborer; four days prior to admission, was suddenly attacked with paralysis of left side. The previous occurrence of an apoplectic stroke, could not be ascertained. Complained of pain in right side of head; sensibility of paralyzed parts not affected.

No. IV, aged 47, laborer; previous condition was not ascertained; when admitted, there was partial paralysis of left upper extremity.

No. V, aged 42, mason; father and brother had suffered from attacks of apoplexy; two years ago, fractured his skull, by a fall; was paralyzed a couple of months, but recovered perfectly the use of his limbs; this was his first attack of apoplexy, — was suddenly prostrated as though he had been struck; lost consciousness; entered hospital, paralyzed on right side.

No. VI, aged 78, butcher; has suffered much from rheumatism; delirious when admitted; previous condition could not be determined; paralyzed on left side; sensibility not affected.

In three of these cases, the previous occurrence of apoplexy could not be determined; but it is fair to presume that it had existed.

In one case, paralysis existed before the loss of consciousness; in another case, the patient was suddenly deprived of his consciousness, of sensation, and of motion; and in still another case, the occurrence of apoplexy was probable. We find, then, in these cases, exemplifications of two of the three different ways in which apoplexy is said to take place: it may be sudden, as in one of the above cases, and it may be preceded by partial paralysis, as occurred in another of these cases. All of these patients were paralyzed to a greater or less degree, on one side, when admitted; the left side being affected in four cases, and the right side in two.

The different forms of apoplexy may terminate in perfect recovery, which is rare; in death, which is common; and in partial or incomplete recovery, which is frequent. The patient usually emerging from the state of coma, with his intellect more or less perverted, the power of motion more or less impaired, and the sensibility to a greater or less degree affected.

The hemiplegia was perfect in none of these cases; some degree of motion was preserved in the extremities, or in the respiratory muscles of the thorax, or in the muscles of the eye, in each one of these cases, although the power of locomotion was lost in every one of them.

The corrugator and orbicular muscles were motionless, in one case, on the affected side; in another case, incompletely so.

The intercostal muscles, on one side, were paralyzed in a single case.

In only two of the six cases, were the extremities perfectly motionless; in the others, there was slight motion in the affected limbs.

In all the cases, the tongue was directed to the affected side; the mouth and the uvula—in every case in which it was observed—were drawn toward the healthy side.

The sensibility of the paralyzed side consequent to an apoplectic stroke, may be extinguished or diminished or perverted. In most of the foregoing cases, it was not materially affected. The reflex movements were readily excited in every patient in whom the experiment was tried. The mental conditions of these patients, after the apoplectie attack had passed away, were quite different. It has been asserted, that—after an apoplectic stroke—the faculties of the mind are more or less changed: the serious person, becoming humorous; and the brave man, a coward. In the above patients the mental condition, prior to their illness, could not very well be determined. But, there were in the ward, at one period, three hemiplegic patients, exhibiting strikingly different conditions of the intellect: one was imbecile; another serious, or depressed; and another witty and humorous.

Much has been said by writers, on the predisposing causes of apoplexy; and various circumstances relative to the patient are generally supposed to favor the production of this disease. Thus, apoplexy is thought to be much more frequent in those classes of persons whose ancestors have suffered from this disease, "in those who possess a peculiar conformation of body," as short and stout individuals, and in those who have passed the prime of life.

In only one of the above cases, the patient's immediate relatives had suffered from apoplectic strokes; two patients possessed the above-mentioned conformation of body; and four of the six patients were attacked at an earlier period of life than usually is the case. They were all laboring men; and some of them accustomed to indulge in spirituous liquors; two had previously sustained severe injuries of the skull, followed by affections of the brain.

We pass now to the consideration of those symptoms which are supposed to indicate the effects produced on the brain, by the presence of a clot of blood. In all those cases of hemiplegia due to the rupture of a cerebral vessel, blood is effused in one of three situations; it is poured out into the ventricles, or between the investing membranes, or into the substance of the brain itself: the latter being altogether the most common. The blood when effused into the brain substance, undergoes changes very similar to those which occur in any other organ, under like conditions. The blood coagulates, and the clot undergoes a variety of changes in different cases. The clot may be gradually, but completely absorbed. Many pathologists state, that—besides absorption of the clot—it may sometimes become organized. Ofter, however, the clot acts like a foreign body, and produces inflammation in the surrounding structure, which may be just sufficient to isolate the clot, or which may be so great as to result in the formation of pus. But there is always more or less irritation or inflammation, produced by the effusion of any amount of blood and the presence of the clot consequent to this effusion, in the substance of the brain. In several of these cases certain symptoms were manifested soon after the apoplectic attack had passed away, which tend to show that the clot of blood was producing more or less disturbance in the brain.

In Case, No. II, the patient was attacked on the 7th of July; on the 9th, he entered the hospital; he was placed on the use of diffusible stimulants and nourishing diet. He continued to improve every day after his admission; his intellect continued clear; he began to have some control over the lower affected extremity; his pulse was 64, and small.

On the 17th of July, *ten days* after date of attack, he manifested some aberration of mind, and the pulse rose to 116. On the night of the 17th, he was delirious, and singultus appeared; pulse 104, and quite feeble.

On the morning of the 18th, complained of slight pain in the head, near vertex in both sides. An opiate was given in place of the stimulant. This patient continued delirious at night, for some weeks.

In Case, No. III, the attack occurred on the 16th of May; admitted with hemiplegia, on the 20th. He seemed to be in a lethargic condition; his intellect was dull; he could tell his age, and how long he had been sick. Pulse small and numbering 72.

On the 22d, *six days* after date of attack, he manifested active delirium, which commenced during the night; he was talking constantly, and making efforts to get out of bed. His aspect was bright, instead of the previous lethargic condition; he was animated: answered questions quickly, but incoherently. Pulse 72. Passed evacuations in bed, involuntarily. Could move slightly the paralyzed limbs. Took his nourishment readily. On his admission, was placed on the use of brandy; various narcotics were added to the treatment; and on June 6th, he reports free from delirium, and very much improved.

Case, No. V, was attacked on night of March 27th; admitted to medical ward on April 5th. Has had no delirium; sleeps well at night; mind clear; pulse 80; appetite good; bowels constipated. A dose of magnes. sulph. was given, and patient placed on use of Huxham's tinct.

April 8th, *twelve days* after date of attack, this patient manifested symptoms of active delirium this morning: delirium during night. He talks incoherently, is irritable and excited by questions addressed to him. He is retained in bed with difficulty. His face is flushed; pulse 100. Brandy was administered.

On the 9th, no delirium during the day, some at night; mind clearer; pulse 76.

In this case, it is to be borne in mind, that the patient had abstained from liquor for five months prior to attack.

No. VI was delirious when admitted; no previous history could be ascertained; he was hemiplegic. Delirium was manifested by incoherent talking and by efforts to get out of bed, mcre marked at night.

In three of these cases, it is well to observe the condition of the patients prior to the appearance of the delirium; and then, to notice the sudden development of delirium, its character, the increased frequency of the pulse, and the result of the treatment.

Only one of these six patients died; this patient, after a protracted illness, succumbed from extreme prostration. The others recovered very slowly, and in a measure the use of the affected limbs. There was much indisposition in two or three of these cases, to make any efforts to get out of bed, sometime after they had regained sufficient strength and ability to move the paralyzed members. These patients had to be lifted out of bed, and various measures resorted to before they could be induced to help themselves.

### *Case of Pseudo-apoplexy.*

James Williams, aged 56 years, admitted, October 10th. When admitted he was incompetent to give any account of his symptoms or history. His eyes were open; his expression was vacant; and he did not attempt to reply to questions. The skin was cool; the pulse small and not accelerated. The persons who brought him to the hospital could give no account of him, except that he was found in his present condition. He made no effort to walk; he required to be brought in and deposited on the bed, without the least exertion on his part. Respiration normal. Brandy and nourishment were alone prescribed.

The next morning he manifested some consciousness, but appeared idiotic.

He smiled when questions were put to him, but said nothing. The following day, improvement was more marked; said he was perfectly well. Could give no account of his attack. From that time he daily improved, and is now (Oct. 21st) walking about the wards, and out of doors; he makes no complaint; appetite is good; his aspect is now intelligent; he replies to questions correctly: says he had no recollection of his attack, nor of his having been brought to the hospital; says he was here two days before he came to himself; says that nothing is the matter with him but weakness.

This patient has the *arcus senilis* well marked, more so on the right than left side: in the right eye it extends nearly around the cornea; in the left, it is limited to the upper half of the circle. The pulse is 80, and quite feeble; skin cool; capillary congestion on hands. The face is pallid; the heart sounds are very faint; no bellows-murmur ascertained; no impulse seen or felt, except a slight shock seen in the epigastrium.

The patient is a carpenter and joiner; drinks moderately, averaging about half a pint daily. Has never had palpitation; never had a similar attack before. Has always enjoyed good health, except three years ago, when he had intermittent fever.

The treatment has consisted of Huxham's tinct., and full diet.

This patient left the hospital on the 24th, feeling very well; has not been heard from since. (*From Prof. Flint's Record.*)

This case was supposed to be one of fatty degeneration of the heart. The diagnosis of this morbid condition cannot always be determined with accuracy, owing to the absence of any symptom characteristic of the affection. There are several symptoms, however, which — when they occur in the same case — render the existence of this condition of the heart, highly probable. "The diagnosis," says Dr. Stokes, "turns upon three points. The existence of physical signs and symptoms of diminished force of the heart. The occurrence of certain symptoms principally referrible to the brain, which indicate anæmia on the arterial, or congestion on the venous side, of the cerebral circulation. And symptoms referrible to the respiratory function which appear to arise from deficient power in the right ventricle." This disease, he says, is most common after the prime of life. The symptoms indicating its existence may be referred to the nervous, respiratory, and circulatory systems. There was presented in the foregoing case, the most important of these nervous phenomena, namely, the sudden supervention of an apoplectic condition: differing from true or sanguineous apoplexy, in the frequency of the attack, and in the infrequency of consequent paralysis. These attacks are due to deficient arterial supply, or to venous congestion in the brain; the former opinion, according to Dr. S., is the most tenable; the suddenness of the attack and the rapidity of the recovery also, favor this view. There were, however, no prominent symptoms presented, referrible to the respiratory or circulatory system. Dr. Stokes mentions a peculiarity in the respiration, which he has only observed in cases of fatty degeneration of the heart. "It consists in the occurrence of a series of inspirations, increasing to a maximum, and then declining in force and length, until a state of apparent apnœa is established. In this condition the patient may remain for such a length of time as to make his attendants believe that he is dead, when a low inspiration, followed by one more decided, marks the commencement of a new ascending and then descending series of inspirations."

A singular appearance of the cornea, has been noticed by Dr. Quain and Mr. Canton, in persons who have been affected with fatty degeneration of the heart. And this appearance when present, will assist in the diagnosis of the disease. Dr. williams, also, has met with this sign in persons who present symptoms of weakened heart. This is *arcus senilis*, a peculiar opacity of the circumference of the cornea, probably due to fatty degeneration of this structure. This sign was finely exemplified in the above case. Attacks of pseudo-apoplexy, accompanied with feebleness of the heart's action, with the presence of arcus senilis, and followed by the beneficial effects of a stimulating and tonic course of treatment, may be supposed to indicate, pretty certainly, the existence of fatty degeneration of the heart.

